# Joint contributions of metacognition and self-beliefs to uncertainty-guided checking behavior

**DOI:** 10.1038/s41598-021-97958-1

**Published:** 2021-09-24

**Authors:** Axel Baptista, Maxime Maheu, Luc Mallet, Karim N’Diaye

**Affiliations:** 1 Institut du Cerveau et de la Moelle épinière, INSERM U1127, CNRS UMR7225, AP-HP Pitié-Salpêtrière, Sorbonne Université, Paris, France; 2 Département d’Études Cognitives, Institut Jean Nicod, ENS, EHESS, CNRS, PSL University, École Normale Supérieure, 29 rue d’Ulm, 75005 Paris, France; 3 Service de Psychiatrie de l’Enfant et de l’Adolescent, GH Pitié-Salpêtrière Charles Foix, AP-HP, Paris, France; 4 Université de Paris, Paris, France; 5 University Medical Center Hamburg-Eppendorf, Hamburg, Germany; 6 Département Médical-Universitaire de Psychiatrie et d’Addictologie, Université Paris-Est Créteil, DMU IMPACT, Hôpitaux Universitaires Henri Mondor - Albert Chenevier, Assistance Publique-Hôpitaux de Paris, Créteil, France; 7 Department of Mental Health and Psychiatry, Global Health Institute, University of Geneva, Geneva, Switzerland

**Keywords:** Psychology, Human behaviour

## Abstract

Checking behavior is a natural and adaptive strategy for resolving uncertainty in everyday situations. Here, we aimed at investigating the psychological drivers of checking and its regulation by uncertainty, in non-clinical participants and controlled experimental settings. We found that the sensitivity of participants’ explicit confidence judgments to actual performance (explicit metacognition) predicted the extent to which their checking strategy was regulated by uncertainty. Yet, a more implicit measure of metacognition (derived from asking participants to opt between trials) did not contribute to the regulation of checking behavior. Meanwhile, how participants scaled on questionnaires eliciting self-beliefs such as self-confidence and self-reported obsessive–compulsive symptoms also predicted participants’ uncertainty-guided checking tendencies. Altogether, these findings demonstrate that checking behavior is likely the outcome of a core explicit metacognitive process operating at the scale of single decisions, while remaining influenced by general self-beliefs. Our findings are thus consistent with two mechanisms (micro vs. macro) through which this otherwise adaptive behavior could go awry in certain psychiatric disorders such as obsessive–compulsive disorder.

## Introduction

Everyday decisions are often marred by uncertainty^1^. Gathering new evidence through exploration is a useful and rational strategy to reduce the impact of uncertainty on subsequent decisions^2^. One of such exploratory strategies is checking, a procedure through which an agent repeatedly samples from the *same* source of information^3,4^. In a changing environment, checking can serve the useful purpose to update one’s, possibly outdated, internal model about the world^5^; for instance, checking one’s smartphone for possible novel notifications. In spite of its apparent simplicity, what drives checking in humans, and how it is regulated by observer’s uncertainty about the world, remains mostly unknown.

Deciphering the psychological drivers of checking is also key to understand how this, otherwise rational, behavior goes awry in certain pathological conditions such as obsessive–compulsive disorder (OCD). About 80% of OCD patients^6,7^ indeed exhibit excessive checking; not only for anxiety-related, but also neutral, stimuli^8–11^. In line with the hypothesized uncertainty-reducing role of checking, patients typically report that checking reduces their uncertainty about checked options^12^.

One hypothesis we explored here is thus that checking behavior depends on an uncertainty-evaluation, or metacognitive, process^13–17^. Traditional models have decomposed metacognitive processes into two categories: (i) *explicit* metacognition, understood as, at least partially, conscious evaluations and regulations of our own decisions, grounded in reflective processes involving introspection, and which enables humans to share metacognitive representations with other people^18–21^; and (ii) *implicit* metacognition understood as pre-reflective, automatic evaluations and regulations of our own decisions, which does not necessarily reach awareness^18,22^, and which are present in non-verbal young infant^23^ and non-human animals^24^. In explicit metacognition tasks, subjects are often asked to rate their confidence into some past decision^20^. By contrast, in implicit metacognition tasks, subjects do not explicitly rate their confidence but, instead, use it to guide decision-making, such as when choosing between several options from which to get rewarded^25^. In line with this taxonomy, here, we contrasted the contributions of both explicit and implicit metacognitive monitoring sensitivities to uncertainty-guided checking behavior, and explored the possibility that checking might only be related to explicit metacognition.

The propensity to check exhibits a wide spectrum from occasional to excessive forms even in the non-clinical population^26^. Previous studies provide converging evidence that self-beliefs (e.g. cognitive distrust) correlate with a general tendency to check, as assessed by questionnaires, in healthy subjects^27,28^. The mechanisms underlying this association are however still unknown. We further hypothesized that self-beliefs such as the self-reported overall propensity to compulsion and cognitive distrust, which varies across individuals, also in the non-clinical population, would impede uncertainty-guided checking behavior.

To address these questions, we capitalized on previous attempts to reproduce checking behavior in controlled experimental settings^3,4,8–10,29^. Notably, we leveraged cutting-edge experimental psychology paradigms allowing us to manipulate perceptual uncertainty through stimulus difficulty similarly across individuals, independently of overall performance. We then quantified the number of checks for different levels of perceptual uncertainty, and assessed to what extent they related to performance. We also measured the sensitivity of participants’ implicit and explicit metacognition using state-of-the-art measures^30,31^. Finally, we made the bridge with widely used clinical notions by relying on validated questionnaires to assess inter-individual differences in declarative self-beliefs.

We made the following hypotheses: (*H*_**1**_) we predicted that checking reduces participants' uncertainty about checked options; (*H*_**2**_) accordingly, we expected that checking allows individuals to increase task performance: performance should be better after checking than after immediate decisions; (*H*_**3**_) importantly, we predicted that checking behavior depends on an explicit uncertainty-evaluation (explicit metacognitive accuracy) rather than an implicit uncertainty-evaluation (implicit metacognitive accuracy) process; (*H*_**4**_) finally, we expected that, above and beyond inter-individual differences in metacognitive sensitivities, participants’ self-beliefs, such as self-reported compulsivity and low self-confidence, would impede checking behavior even in healthy subjects. In a nutshell, our findings show that uncertainty-guided checking behavior relates to both participants’ (explicit, but not implicit) metacognitive sensitivity as well as to self-beliefs.

## Results

We report results for a total of 28 human healthy participants who were asked to rate the orientation (left or right) of random dot motion (RDM) stimuli. Difficulty levels (i.e. motion coherence) were adjusted at the single participant level thanks to an initial calibration phase (see Methods), to prevent inter-individual differences in visual perception to affect the results.

### Checking behavior

**Figure 1:**
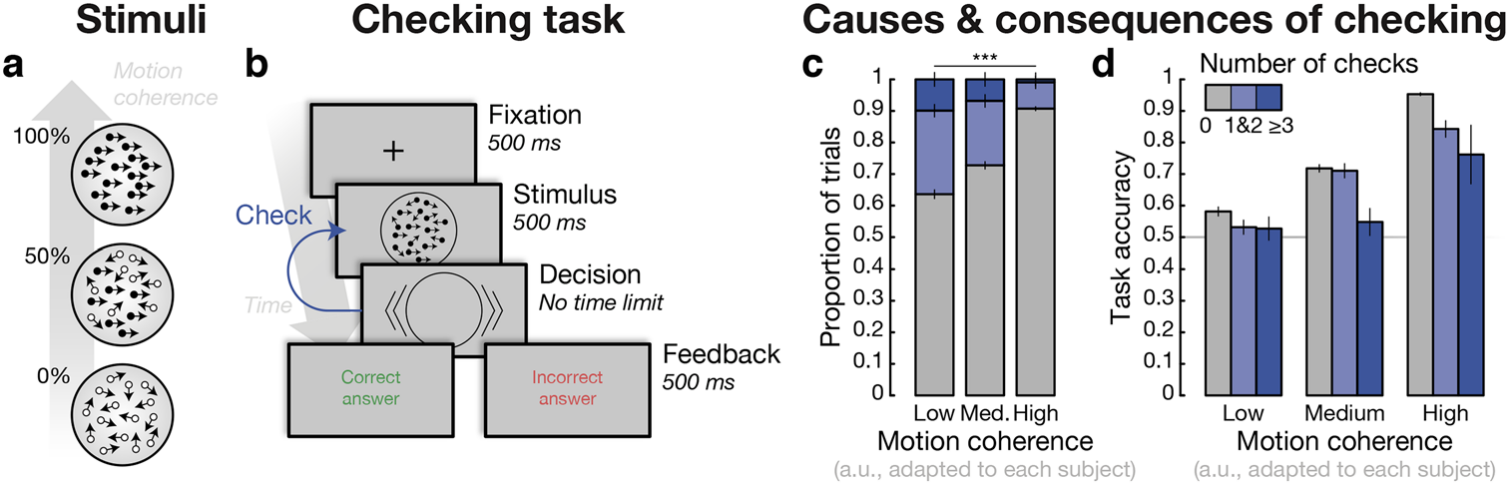
Causes and consequences of checking behavior. (**a**) *RDM stimuli*. Stimuli used throughout the study were random dot motion (RDM) stimuli whose coherence level was manipulated. Participants were asked to judge the direction of motion (either left or right). (**b**) *Checking task*. Representative trial of the checking task: before committing to the decision, participants were allowed to replay the RDM stimulus an infinite number of times. Feedback was delivered at the end of the trial. (**c**) *Increased checking after more difficult stimuli*. Number of checks according to motion coherence (i.e. difficulty, in arbitrary units, a.u.), which is expressed in three terciles for the sake of averaging over participants (who have different perceptual sensitivity). (**d**) *Decreased performance after checking.* Accuracy of orientation judgments as a function of motion coherence and number of checks. Same binning method as in (**c**). Error bars correspond to standard error of the mean.

Participants performed a checking task in which they were allowed to replay (an infinite number of times) the RDM stimulus before committing to the decision orientation (see Methods and Fig. 1a,b). We first assessed fluctuations of checking behavior, how checking is regulated by stimulus difficulty and whether it improves subsequent performance.

Firstly, in order to evaluate the impact of stimulus difficulty on checking (*H*_**1**_), we used a Poisson mixed effect model explaining trial-by-trial fluctuations in the number of checks (hereafter referred to as *ℳ*_1_; see Methods and Table S1). We found that participants increased their checking frequency for more difficult stimuli (*b* = 0.90, *P* < 2 × 10^–16^; see Fig. 1c), in line with the hypothesis that checking aims at decreasing participants’ uncertainty about future decisions (*H*_**1**_).

Secondly, in order to evaluate whether checking behavior improved subsequent performance (*H*_**2**_), we used a multivariate logistic mixed-effect model explaining trial-by-trial fluctuations in RDM task performance (hereafter referred to as *ℳ*_2_; see Methods and Table S2). Contrary to what we expected, we found that checking decreased performance on RDM stimuli of matched difficulty levels (*b* = –0.26, *P* = 4.56 × 10^–8^; see Fig. 1d). Furthermore, we found that the detrimental effect of checking on performance decreased with stimulus difficulty (number of checks by stimulus difficulty significant and positive interaction; *b* = 0.20, *P* = 6.27 × 10^–5^): checking more strongly reduced performance for high motion coherence RDM stimuli (i.e. easy trials). Although checking was promoted by perceptual uncertainty (*H*_**1**_), it thus did not appear to reduce the impact of perceptual uncertainty on subsequent decisions (*H*_2_); we later discuss possible reasons for this finding (see Discussion).

### Contribution of metacognition to checking

Showing that our experimental settings induced uncertainty-guided fluctuations in checking behavior, we now turn to one of our study goals: assessing the contribution of inter-individual differences in metacognitive sensitivities in the tendency of participants to check (*H*_**3**_). In particular, we assessed a possible differential effect between implicit and explicit metacognitive sensitivities in the promotion of checking and its regulation by perceptual uncertainty.

**Figure 2:**
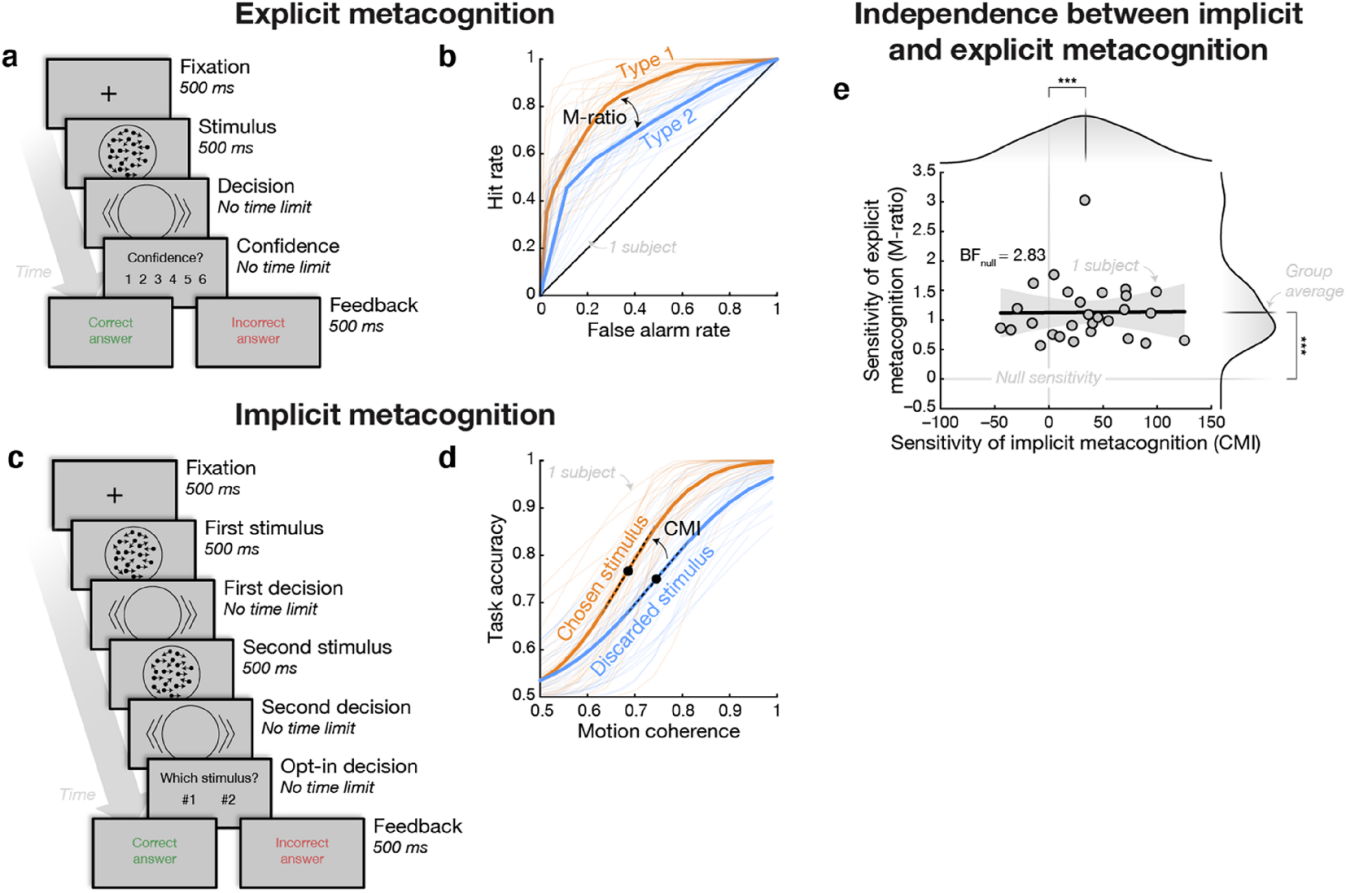
Independence of implicit and explicit metacognitive sensitivities. (**a**) *Explicit metacognition task*. Representative trial of the explicit metacognition task: after reporting their orientation judgment, participants were asked to report their confidence in this orientation judgment on a scale from 1 (guess) to 6 (certainty). Feedback was delivered at the end of each trial. (**b**) *Sensitivity of explicit metacognition*. The sensitivity of explicit metacognition was measured using the M-ratio, which quantifies the amount of decision information available to the confidence judgment, here depicted as the difference between type 1 (decision) and type 2 (confidence) ROC curves^35^. (**c**) *Implicit metacognitive task*. Representative trial of the implicit metacognition task: after reporting their orientation judgment for two successive RDM stimuli, participants were asked to report the stimulus for which they judged more likely to be correct. Feedback on the chosen stimulus was delivered at the end of each trial. (**d**) *Sensitivity of implicit metacognition*. The sensitivity of implicit metacognition was measured as the difference in slopes between psychometric functions corresponding to the chosen minus discarded stimuli, divided by the average slope across both conditions; a measure we refer to as CMI^25,30^. (**e**) *Independence between explicit and implicit metacognition*. Metacognitive sensitivities were estimated for each participant separately in the explicit and implicit metacognition tasks. Measures are not correlated across participants suggesting they relate to independent metacognitive processes (see Table S3). Stars indicate significance: *P < 0.05, **P < 0.01, and ***P < 0.001.

In a well-established explicit metacognition task (see for example^32^), participants were asked to explicitly rate their confidence (on a scale ranging from 1, low confidence, to 6, high confidence) about their orientation judgment (see Fig. 2a). We assessed participants’ explicit metacognitive sensitivity using M-ratio, a well-established measure of (explicit) metacognitive sensitivity which quantify the amount of perceptual information (in signal-to-noise units) that is available to metacognitive processes^33^. Overall, participants had an M-ratio significantly higher than zero (two-tailed one sample Wilcoxon signed rank test; *V*-statistic = 406; *P* value = 4.003 × 10^–6^; see Fig. 2b), thereby showing that confidence judgments were predictive of actual performance.

In an implicit metacognition task, using an original design by Barthelmé and Mamassian^34^ and validated in further studies^25,30,34^, participants had to judge the direction of two successive RDMs, of different difficulty levels (i.e. motion coherence), before being finally asked to indicate the one for which they judged to be more likely correct (see Fig. 2c). We assessed participants’ implicit metacognitive sensitivity by computing the difference in sigmoid slopes (relating stimulus difficulty to performance) between the discarded and the chosen stimulus (divided by the average slope across both conditions), hereafter referred to as the confidence modulation index (CMI)^25,30^. Participants’ CMI was significantly higher than zero (two-tailed one sample Wilcoxon signed rank test; *V*-statistic = 351; *P* value = 3.814 × 10^–4^; see Fig. 2d), thereby showing that participants were able to leverage their metacognitive knowledge to improve decision-making.

**Figure 3:**
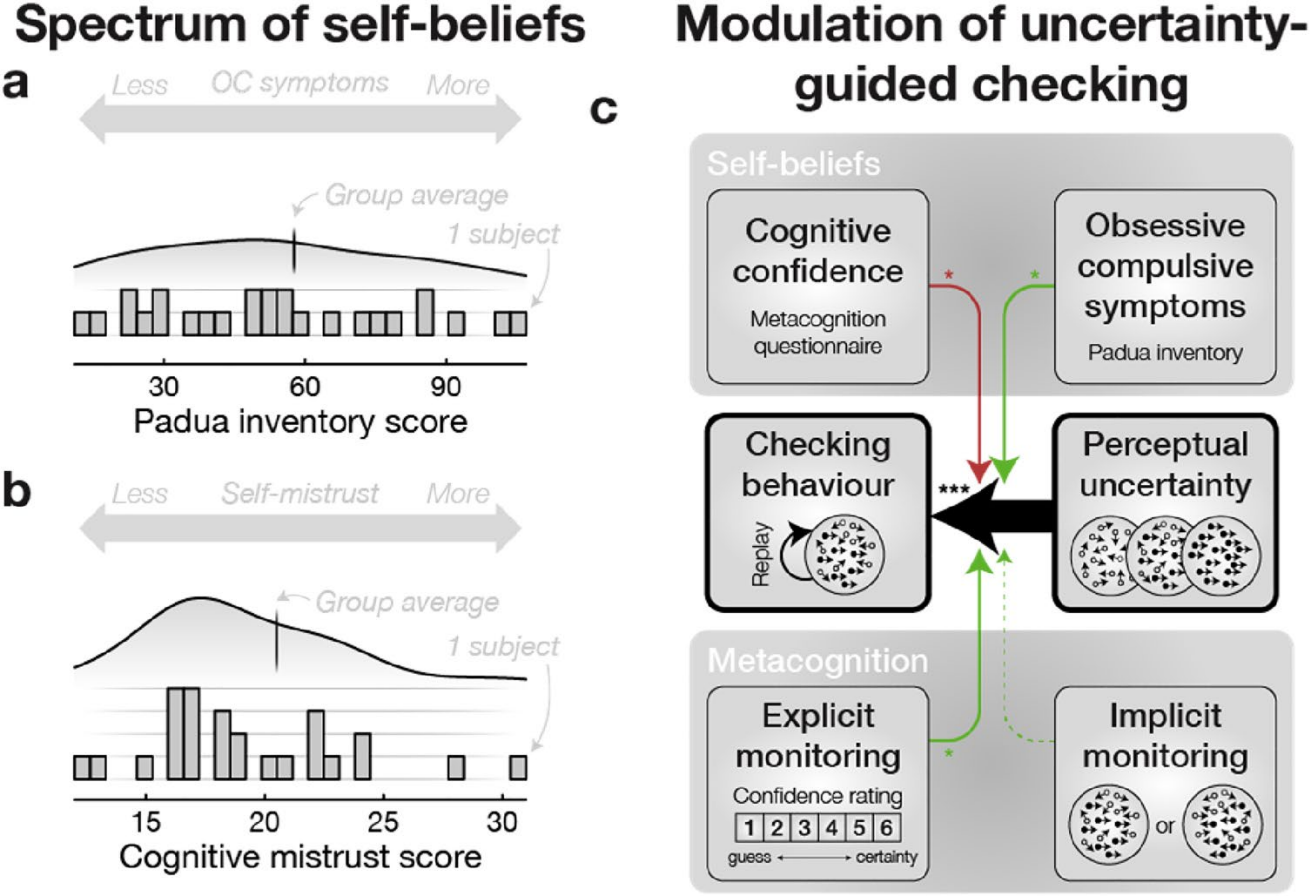
Modulation of checking uncertainty-guided behavior. (**a**/**b**) *Distribution of participants on the Padua inventory/cognitive mistrust subscales*. Participants spread over a large spectrum of self-beliefs, which replicated previous findings^28,36,37^ (see Methods). (**c**) *Modulation of uncertainty-guided checking*. Arrow size are proportional to *z*-scores from the Poisson model of the number of checks (*ℳ*_1_, see Methods). Arrow color denotes the direction of the effect (green: positive, red: negative). Stars indicate significance: *P < 0.05, **P < 0.01, and ***P < 0.001.

We then assessed the independence between implicit vs. explicit metacognitive sensitivities by correlating them across participants. We found no significant correlation (*r* = 0.04, *P* = 0.853, BF_null_ = 2.83; see Fig. 2e & Table S3), thereby suggesting they reflect distinct (meta)cognitive processes and, possibly, relate differently to checking behavior. Accordingly, we also found a dissociation in the contributions of explicit vs. implicit metacognitive sensitivities to uncertainty-guided checking behavior (*H*_**3**_). On the one hand, the Poisson mixed effect model explaining trial-by-trial fluctuations in the number of checks (*ℳ*_1_) revealed a significant positive interaction between M-ratio and stimulus difficulty (*ℳ*_1_; *b* = 0.08, *P* = 0.028; see Fig. 3c), showing that checking was driven by perceptual uncertainty more strongly in participants with higher explicit metacognitive sensitivity. Conversely, the interaction between CMI (i.e. implicit metacognitive monitoring ability) and stimulus difficulty was negative and non-significant (*ℳ*_1_; *b* = –0.05, *P* = 0.210; see Fig. 3c). We then confirmed that the differential modulation of implicit vs. explicit metacognitive sensitivities on uncertainty-guided checking behavior was significant using a likelihood ratio test (LRT) between the original model (*ℳ*_1_) versus a restricted model which assumes the equality between the regression coefficients of the interactions of interest (i.e. interactions between CMI and stimulus difficulty, and M-ratio and stimulus difficulty; LRT: chi-square = 5.88, 1 df, *P* = 0.015). This analysis thus confirmed that checking is more strongly driven by perceptual uncertainty in participants with elevated explicit, but not implicit, metacognitive monitoring ability (*H*_**3**_).

### Contribution of self-beliefs to checking

We hypothesized that, above and beyond inter-individual differences in metacognitive sensitivities, participants’ self-beliefs, such as self-reported compulsivity and self-confidence, would modulate checking behavior even in our non-clinical population (*H*_**4**_). To investigate this, we asked participants to answer questionnaires at the end of our experiment.

First, we measured the declarative lack of confidence in various cognitive domains (e.g. “my memory can mislead me at times”) using the cognitive mistrust subscale of the Metacognition Questionnaire^28^ (see Fig. 3b). This subscale was of particular interest here as a long series of studies (see^27^ for a review) consistently showed that persons suffering from compulsive checking typically exhibit higher levels of cognitive mistrust. Here, we tested, in particular, the hypothesis that cognitive mistrust may decrease the sensitivity of checking behavior to perceptual uncertainty. Accordingly, we found that cognitive mistrust significantly reduced the effect of stimulus difficulty on checking behavior (*ℳ*_1_; cognitive mistrust by stimulus difficulty significant negative interaction; *b* = –0.09, *P* = 0.039; see Fig. 3c).

Second, we measured self-reported obsessive–compulsive symptoms (OCS) using the Padua inventory scale^36^ which quantifies self-reported obsessions and rituals across four dimensions: mental control, checking, contamination obsessions and anxiety about motor control (see Fig. 3a). The Padua Inventory scale was slightly correlated with the cognitive mistrust subscale across participants (*r* = 0.42, *P* = 0.026; see Table S3), as previously observed^28^. We found, however, that, contrary to the cognitive mistrust subscale, the self-reported obsessive–compulsive symptoms increased the effect of stimulus difficulty on checking behavior (*ℳ*_1_; Padua inventory by stimulus difficulty significant positive interaction; *b* = 0.10, *P* = 0.019; see Fig. 3c).

## Discussion

The aim of the present study was to investigate the psychological drivers of checking behavior in humans, in particular its regulation by environmental uncertainty. Our results provide evidence that human checking behavior is the outcome of an uncertainty-evaluation, or metacognitive, process (*H*_**1**_). Indeed, participants increased their checking frequency for more difficult stimuli, and thus more uncertain settings^38^. This result is in line with past findings in non-human primates^4,39^ which demonstrate that checking adaptively aims at decreasing one’s uncertainty about future decisions.

Contrary to what was expected, checking had a detrimental effect on performance: accuracy was worse after checking than after immediate decisions (*H*_**2**_). A first possible explanation of this unexpected result relates to the psychophysical properties of the RDM stimuli used in this study: although the difficulty of the RDM stimulus was kept constant across all replays (if any), each replayed RDM differed in the exact spatial locations of the dots. A possibility is that participants expected to see the exact same RDMs across all replays. The incongruence of replayed RDMs with subject’s expectations may have impaired subsequent decision-making processes. To investigate this hypothesis, future studies could manipulate the extent to which stimulus replays vary from the original stimulus and compare the impact on performance. Worth noting, above a given degree of stimulus dissimilarity, it is likely that the cognitive process at stake will relate more to ‘information sampling’ than genuine ‘checking behavior’^11^. Further theoretical work is highly needed to devise the conceptual delineations of those processes. A second, perhaps more interesting, explanation for the detrimental effect of checking on subsequent performance is that repeated checking of the RDM stimuli may hinder (perceptual) decision-making processes. This possibility echoes the results of a recent meta-analysis which revealed that repeated checking reduced memory accuracy of visual stimuli across experiments, in addition to the well-known reduction in memory confidence^40^. The mechanisms underlying this reduction in memory performance are not yet known. A possibility is that, in our study, the diminished perceptual decision-making performance relies on similar mechanisms. Further studies are necessary to replicate our unexpected result, and to investigate its possible underpinnings. Importantly, our results show that although detrimental on average, checking nevertheless remains adaptive in the sense that this detrimental effect seems to decrease with stimulus difficulty, thereby suggesting it is still more adapted to check after more difficult stimuli.

Our study also provides evidence that implicit and explicit metacognitive sensitivities are dissociable (*H*_**3**_) and that they relate differently to uncertainty-guided checking behavior. We found that, in the non-clinical population, uncertainty-guided checking behavior is promoted by a conscious, *explicit* high-level mental representation^18–20^, rather than by an unconscious, *implicit* low-level representation of perceptual uncertainty^18,22^. The role of this dichotomy in shaping checking behavior has received surprisingly little attention despite its important implications for disorders of checking behavior, including OCD. It has been suggested that excessive checking behavior in compulsive checkers corresponds to the pathological automatization of an otherwise deliberate behavior^40–43^. One could thus hypothesize that such an automatization relates to an insufficient contribution of explicit metacognitive processes to uncertainty-driven checking in compulsive checkers. This insufficient contribution could result from an alteration of the ability to explicitly introspect about one’s decisions (an alteration of the so-called metacognitive *monitoring* ability) as suggested by recent studies^44,45^. Another possibility is that the ability to explicitly introspect about one’s decisions is preserved, but the resulting information is not appropriately used for the regulation of checking behavior. To our knowledge such a metacognitive *control* impairment has never been investigated in OCD. In either case, compulsive checkers would lack an explicit, conscious termination cue, and would continue checking regardless of environmental uncertainty.

Over and above the contribution of core metacognitive processes operating at the scale of single decisions, our results suggest that uncertainty-guided checking behavior is also influenced by self-beliefs (*H*_**4**_), including the self-reported propensity to compulsions and cognitive mistrust. Previous studies provide converging evidence that self-reported overall confidence in memory is lowered by repetitive checking in healthy subjects^40^ and that OCD patients have lowered confidence in memory, as well as in other cognitive domains^27^. Nonetheless, the mechanism underlying the typical association between excessive checking and cognitive mistrust is still unknown. Our results bring a step forward in understanding by showing that cognitive mistrust impedes the regulation of checking behavior by perceptual uncertainty. It follows that the alteration of uncertainty-guided checking behavior may mediate the association between cognitive mistrust and obsessive–compulsive symptoms (OCS), a hypothesis which remains to be tested.

Conversely, our results show that beyond cognitive mistrust, self-reported OCS are associated with checking behavior better tuned to environmental uncertainty, operationalised here as stimulus difficulty. This goes against our hypothesis that the propensity to compulsion, conceived as a continuum from non-clinical OCS to OCD^26^, would impede uncertainty-guided checking behavior (*H*_**4**_). A possible explanation for this result is that the relation of compulsivity to uncertainty-driven checking behavior may depend on whether compulsivity falls within the clinical or non-clinical domain. The association between compulsivity and the impairment of uncertainty-guided checking behavior would thus appear clearly only for pathological compulsivity. Future studies in patients with OCD are necessary in order to test these hypotheses. To that regard, our well-controlled experimental setting might be relevant in assessing checking behavior in OCD patients.

Our findings should be relativized given the exploratory nature of our study and its limitations. In particular, we found two unexpected findings which we call for replications: a detrimental effect of checking on performance, and an association between increased self-reported OCS and improved uncertainty-guided checking behavior. A classic but major limitation of our study concerns our sample size. Although we collected a large number of trials to maximize within-subject reliability and sensitivity of our results, we believe it is important to confirm our results in larger sample sizes. We also note that given recent evidence reporting an impact of feedback onto self-reported measures^46^, it is possible that feedback provided in the course of the experiment may have influenced subsequent self-reports, including cognitive distrust and OCS. However, because performance was individually tailored (to ~ 70% accuracy), it is likely that the impact of task feedback onto subjects’ self-beliefs, if any, was uniform across subjects, and thereby could not by itself explain our results. In addition, in previous research, implicit metacognition is assessed in different ways and the ideal behavioural task for its assessment is still debated. Some tasks require subjects to place bets on their decisions or give subjects the possibility to opt out^47^. However, whether these implicit (non-verbal) uncertainty-monitoring tasks are metacognitive is a matter of debate^21,48^. In the present study, the implicit metacognition task required subjects to choose among two stimuli the one they judged the most likely to yield a correct response^25,30,34^. As Mamassian and colleagues showed, when doing so, participants' have to compare two levels of uncertainty related to two successive decisions^34^. This ‘comparison step’ makes the so-called implicit metacognition slightly more complex, possibly involving meta-representations, than the report of a single perceptual uncertainty quantity, which could be explained by entirely first-order processes^48^. Also, it is possible that in individuals’ everyday environment, in particular at home, the metacognitive processes involved in repetitive checking may differ from those elicited in the lab. Accordingly, it has been shown that personalized cues trigger increased responses in OCD, compared to non-specific ones^49^. Our hypotheses should thus also be tested in a more ecological environment, such as at participants’ homes. The development of information and communication technologies, and their recruitment for cognitive tasks, could make it possible to export research outside the laboratory and improve external validity of cognitive models.

In summary, our findings provide evidence for joint contributions of both explicit, but not implicit, metacognitive processes, and self-beliefs, including cognitive mistrust, to checking behavior guided by perceptual uncertainty. Our findings also open unexplored avenues to address and better understand how the otherwise adaptive checking behavior could go awry in OCD.

## Methods

### Participants

Sample size was determined a priori based on past studies of metacognitive sensitivity^25,30^, and constrained by participant availability. We initially aimed for a target sample size of 40 participants, and our initial sample (i.e. before outlier rejection) consisted of 37 healthy volunteers. We tested our hypothesis using multilevel regression models (see below;^50^, for which it remains difficult to perform classic power calculations, mostly because of the multiple sources of variance that must be taken into account^51^). Because the study did not concern biomedical research and no medical intervention was performed, it was, according to French law, waived from getting an approval by an external ethical committee. Yet, an internal review board, the ‘Comité de pilotage du Laboratoire d'Economie Expérimentale de Paris (LEEP)', assessed and approved the nature of the study and procedures, which preserved the full anonymity of the participants with respect to experimental data collection. The study was carried out in accordance with the Declaration of Helsinki^52^. All participants provided informed consent and received 15 euros for participating in the study plus a bonus reward (max = 5 euros) proportional to their performance during the behavioral experiment.

### Behavioral experiment

The behavioral experiment, which lasted for 1h30min, consisted in 4 behavioral tasks, all derived from the well-established random-dot motion (RDM) task^53,54^ (see Fig. 1a). The RDM stimuli consisted of 236 white dots presented for 500 ms. Difficulty was manipulated by varying the degree of motion coherence (i.e. the proportion of dots moving in the same direction). On each trial, the direction of the dots moving coherently was randomly set to left or right. Participants reported the perceived motion direction on a keyboard; no time limit was imposed. Feedback was delivered at the end of each trial. First, participants went through a calibration phase (see below), followed by three tasks which were all explained in advance, and after a training phase of 15 trials was completed. The implicit metacognitive task and the checking task (see below) were intermixed in blocks of 50 trials for a total duration of 50 min, resulting in a mean number of trials of respectively 246.4 (median = 251, sd = 21, min = 204, max = 300) and 218.5 (median = 201, sd = 22.8, min = 201, max = 251). The explicit metacognition task (see below) was presented in one single block at the end of the experiment for a total duration of 15 min, resulting in a mean number of trials of 113.3 (median = 117.5; sd = 17.1; min = 79; max = 142). Data from all tasks are available at https://doi.org/10.17605/OSF.IO/2TAC4.

#### Calibration phase

Difficulty of RDM stimuli (i.e. motion coherence) was adjusted so as to evoke the same performance level across participants. This allowed to estimate inter-individual differences in metacognitive sensitivities independently of differences in low-level visual perception, which otherwise typically contaminate metacognitive measures^20,31^. For that aim, prior to the experiment, we estimated, for each participant, psychometric curves (inverse logit function) relating motion coherence levels to orientation judgment accuracy in a set of 120 RDM stimuli. During calibration, coherence levels were chosen online so as to maximally improve the model evidence of the psychometric function thanks to an adaptive variational Bayesian design optimization algorithm^55^. We discarded 9 participants from the analyses, for which either (i) the slope of the psychometric curve derived from the calibration task was more than 2 SE below group's mean slope, or (ii) the stimulus coherence level at the inflexion point of the psychometric curve was more than 2 SE above group average. Our final sample consisted of 28 participants aged 19 to 29 years old (mean = 23.6, sd = 3.0).

#### Checking task

Participants were asked to judge the orientation of a RDM stimulus they could replay an infinite number of times. Difficulty of the first RDM stimulus was drawn at random, and kept constant for the replayed RDM stimuli (if any). Dots’ spatial locations varied between successive presentations of the RDM stimulus.

#### Implicit metacognitive monitoring task

Participants were asked to judge the orientation of two RDM stimuli presented in succession, then asked to choose the stimulus for which they felt the most likely to be correct (see Fig. 2c)^25,30,34^. Difficulty of the two RDM stimuli were varied independently. Again, using participant-specific psychometric curves estimated during the calibration task, we chose coherence levels such that participants' performance ranged from 50 to 100% correct.

#### Explicit metacognitive monitoring task

Participants were asked to judge the orientation of a RDM stimulus as well as a to report a post-decisional confidence judgment^20,31^ using a discrete scale ranging from 1 (guess) to 6 (certainty; see Fig. 2a). Prior to the experiment, participants were instructed to use the entire length of the confidence scale. They used arrows keys to move a square indicating the selected confidence level; until they reached their desired confidence level, at which point they pressed the space bar to confirm their rating. The cursor starting point was set to a random position between 1 and 6. Using participant-specific psychometric curves estimated during the calibration task, we chose coherence levels such that participants' performance ranged from 50 to 100% correct.

### Measures of metacognitive sensitivity

#### Implicit metacognition

We used the methodology previously implemented by de Gardelle and colleagues^25,30^. We fitted two psychometric curves (inverse probit function) describing the proportion of correct responses as a function of RDM difficulty separately for chosen versus discarded stimuli. We then quantified the so-called confidence modulation index (CMI) as the difference in sensitivity (i.e. slope) between the two psychometric curves, divided by the average sensitivity across of the two psychometric curves^25,30,56^.

#### Explicit metacognition

We used the M-ratio^31,33,35^, a well-established measure of metacognitive efficiency which maps the sensitivity of participants' confidence judgments about their performance to their actual objective performance, independently of task difficulty.

### Questionnaires

Participants answered the ‘*general cognitive mistrust*’ and ‘*obsessive–compulsive symptoms*’ questionnaires.

#### Cognitive mistrust

The level of cognitive mistrust was assessed with the French version of the *MetaCognition Questionnaires*^28^. Participants’ mean cognitive mistrust subscale score (mean = 20.5, sd = 6.3) was similar to MCQ first validation study scores in a non-clinical population (mean = 17.9, sd = 6.0 for male, mean = 17.8, sd = 5.4 for female)^37^, as well as the MCQ French validation study scores (mean = 19.0, sd = 6.3 for male, mean = 18.4, sd = 5.8 for female) in a non-clinical population^28^.

#### Obsessive Compulsive Symptoms (OCS)

OCS were assessed using the Padua Inventory^36^, a 60-items Likert scale which measures obsessions and rituals (motor or mental). Participants’ mean Padua inventory score (mean = 57.6, sd = 28.8) was similar to Sanavio et al.’s study with non-clinical participants’ (mean = 53.6, sd = 27.7 for male, mean = 62.5, sd = 29.2 for female)^36^.

### Statistical models

Models were implemented using the lme4^57^ package in R version 3.4.1^58^. Regression coefficients (*b*) and their statistics (*z*-scores and *P*-values), calculated using the Wald method^57^, as well as bootstrapped 95% confidence intervals, are reported (see Table S1 & S2). All predictors were standardized. For each regressor in the models, we checked for multicollinearity using the variance inflation factor (VIF; Shieh and Fouladi^59^). A VIF smaller than 3 indicates that collinearity is not an issue^60^.

*Model #1* (*ℳ*_1_)**.** The model relates trial-by-trial fluctuations of the number of checks to trial-by-trial changes in RDM difficulty, as well as trait-level predictors including implicit and explicit metacognitive sensitivities (i.e. CMI and M-ratio, respectively), questionnaires (i.e. Padua Inventory scale and MCQ’s cognitive mistrust subscale) as well as interactions between these predictors and trial-level stimulus difficulty. Practically, the model is a Poisson generalized linear mixed model (*N*_obs_ = 6119; *N*_subj_ = 28) with participants as random intercept, and an observation level random effect used to account for the overdispersion of the dependent variable, as recommended by Harrison^61^. We computed VIF for all regressors and found an average VIF of 1.2 (sd = 0.1, median = 1.1, max = 1.4), demonstrating collinearity was not an issue in our model. This model allowed us to test hypothesis *H*_**1**_, *H*_**3**_, and *H*_**4**_*.*

*Model #2* (*ℳ*_2_). The model relates trial-by-trial fluctuations in the accuracy of orientation judgments to trial-by-trial fluctuations in number of checks, RDM difficulty as well as trait-level predictors including implicit and explicit metacognitive sensitivities (i.e. CMI and M-ratio, respectively), Padua Inventory and MCQ’s cognitive mistrust subscale, as well as interactions between these predictors and trial-level number of checks. Practically, the model is a logistic linear mixed model (*N*_obs_ = 6119; *N*_subj_ = 28) with participants as random intercept. Again, we found an average VIF of 1.13 (sd = 0.7, median = 1.1, max = 2.8), demonstrating collinearity was not an issue in our model. This model allowed us to test hypothesis *H*_**2**_.

## Open data

All data from this study have been made publicly available via the *Open Science Framework* and can be accessed at https://doi.org/10.17605/OSF.IO/2TAC4.

## Supplementary Information

Supplementary Information.
