# Supplementary material for: Joint contributions of metacognition and self-beliefs to uncertainty-guided checking behavior
Source: Sci Rep. 2021 Sep 24;11:19017. doi: 10.1038/s41598-021-97958-1 (PMC8463683; doi:10.1038/s41598-021-97958-1)
Supplement: Supplementary file 1 — Supplementary Information. [file 41598_2021_97958_MOESM1_ESM.docx]

# Supplementary information

**Table S1** | **Poisson mixed model (***ℳ*_1_**) of the number of checks**

Regression coefficients of main and first-order interaction effects.

|  | **Estimate (SE)** | **Bootstrapped CI**  **[2.5%, 97.5%]** | ***z*-value** | ***p*-value** |
| --- | --- | --- | --- | --- |
| **Main effects** | | | | |
| Intercept | –2.01  (0.10) | [–2.34, –1,89] | –20.86 | < 2 × 10^–16^ *** |
| M-ratio | 0.17  (0.09) | [–4.54 × 10^–3^, 0.33] | 1.95 | 0.051 |
| CMI | 0.18  (0.09) | [–1.22 × 10^–3^, 0.35] | 1.94 | 0.053 |
| Cognitive mistrust | 0.10  (0.10) | [–0.10, 0.27] | 1.00 | 0.316 |
| Padua inventory | –0.03  (0.10) | [–0.21, 0.16] | –0.28 | 0.777 |
| Difficulty | 0.90  (0.04) | [0.81, 0.95] | 23.57 | < 2 × 10^–16^ *** |
| **Interaction effects** | | | | |
| M-ratio × Difficulty | 0.08  (0.04) | [2.27 × 10^–2^, 0.16] | 2.20 | 0.028 * |
| CMI x Difficulty | –0.05  (0.04) | [–0.12, 2.83 × 10^–2^] | –1.25 | 0.210 |
| Cognitive mistrust × Difficulty | –0.09  (0.04) | [–0.16, – 2.41 × 10^–4^] | –2.06 | 0.039 * |
| Padua inventory × Difficulty | 0.10  (0.04) | [1.60 × 10^–2^, 0.18] | 2.35 | 0.019 * |

**Table S2** | **Logistic mixed regression model (***ℳ*_2_**) of performance**

Regression coefficients of main and first-order interaction effects.

|  | **Estimate (SE)** | **Bootstrapped CI**  **[2.5%, 97.5%]** | ***z*-value** | ***p*-value** |
| --- | --- | --- | --- | --- |
| **Main effects** | | | | |
| Intercept | 1.41  (0.07) | [1.28, 1.54] | 21.23 | < 2 × 10^–16^ *** |
| Checking | –0.26  (0.05) | [–0.36, –0.16] | –5.47 | 4.56 × 10^–8^ *** |
| Difficulty | –1.12  (0.04) | [–1.21, –1.04] | –25.59 | < 2 × 10^–16^ *** |
| M-ratio | –0.05  (0.06) | [–0.17, 6.96 × 10^–2^] | –0.86 | 0.389 |
| CMI | 0.05  (0.06) | [–8.32 × 10^–2^, 0.16] | 0.77 | 0.440 |
| Padua inventory | 0.12  (0.07) | [–2.23 × 10^–2^, 0.26] | 1.77 | 0.076 |
| Cognitive mistrust | –0.03  (0.07) | [–0.17, 0.10] | -0.42 | 0.672 |
| **Interaction effects** | | | | |
| Checking × Difficulty | 0.20  (0.05) | [9.09 × 10^–2^, 0.29] | 4.00 | 6.27 × 10^–5^ *** |
| Checking x M-ratio | 0.005  (0.03) | [–6.19 × 10^–2^, 6.57 × 10^–2^] | 0.17 | 0.867 |
| Checking × CMI | 0.02  (0.03) | [–4.33 × 10^–2^, 9.23 × 10^–2^] | 0.58 | 0.562 |
| Checking × Padua inventory | –0.03  (0.03) | [–9.87 × 10^–2^, 4.54 × 10^–2^] | –0.81 | 0.419 |
| Checking × Cognitive mistrust | –0.07  (0.04) | [–0.15, –3.53 × 10^–3^] | –2.00 | 0.045 * |

**Table S3** | **Correlation matrix using spearman method with listwise deletion**

|  | CMI | Cognitive mistrust | Padua Inventory score |
| --- | --- | --- | --- |
| M-ratio | *r* = 0.04  (*p* = 0.853) | *r* = 0.03  (*p* = 0.880) | *r* = –0.16  (*p* = 0.416) |
| CMI |  | *r* = –0.16  (*p* = 0.408) | *r* = –0.10  (*p* = 0.627) |
| Cognitive mistrust |  |  | *r* = 0.42  (*p* = 0.026*) |
